# Supplementary figures and images for: A novel endocast technique providing a 3D quantitative analysis of the gastrovascular system in Rhizostoma pulmo: An unexpected through-gut in cnidaria
Source: PLoS One. 2022 Aug 4;17(8):e0272023. doi: 10.1371/journal.pone.0272023 (PMC9352040; doi:10.1371/journal.pone.0272023)

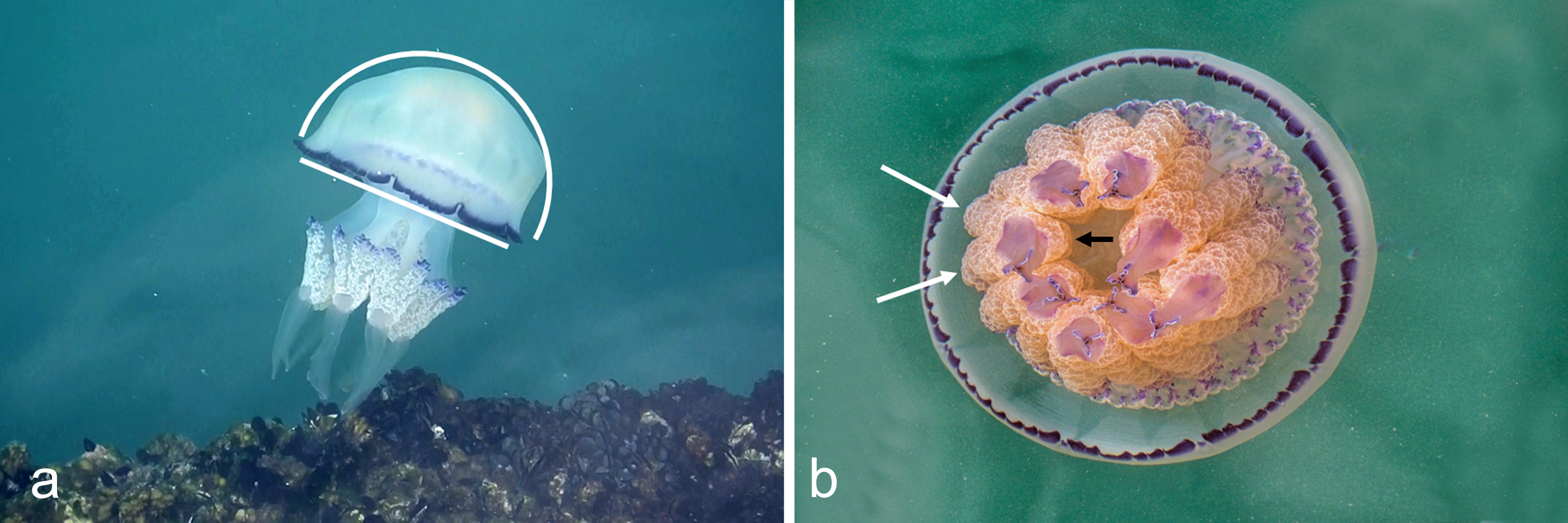

Supplement: S1 Fig — (a) Semicircle indicates the real umbrellar diameter, straight line indicates the apparent diameter. (b) Upside-down specimen showing the three-winged pattern of the oral arms. White arrows indicate the two external wings, black arrow the internal one (Photo courtesy of Paolo Coretti). Both specimens have an apparent diameter of about 30 cm. (TIF) [file pone.0272023.s001.tif]

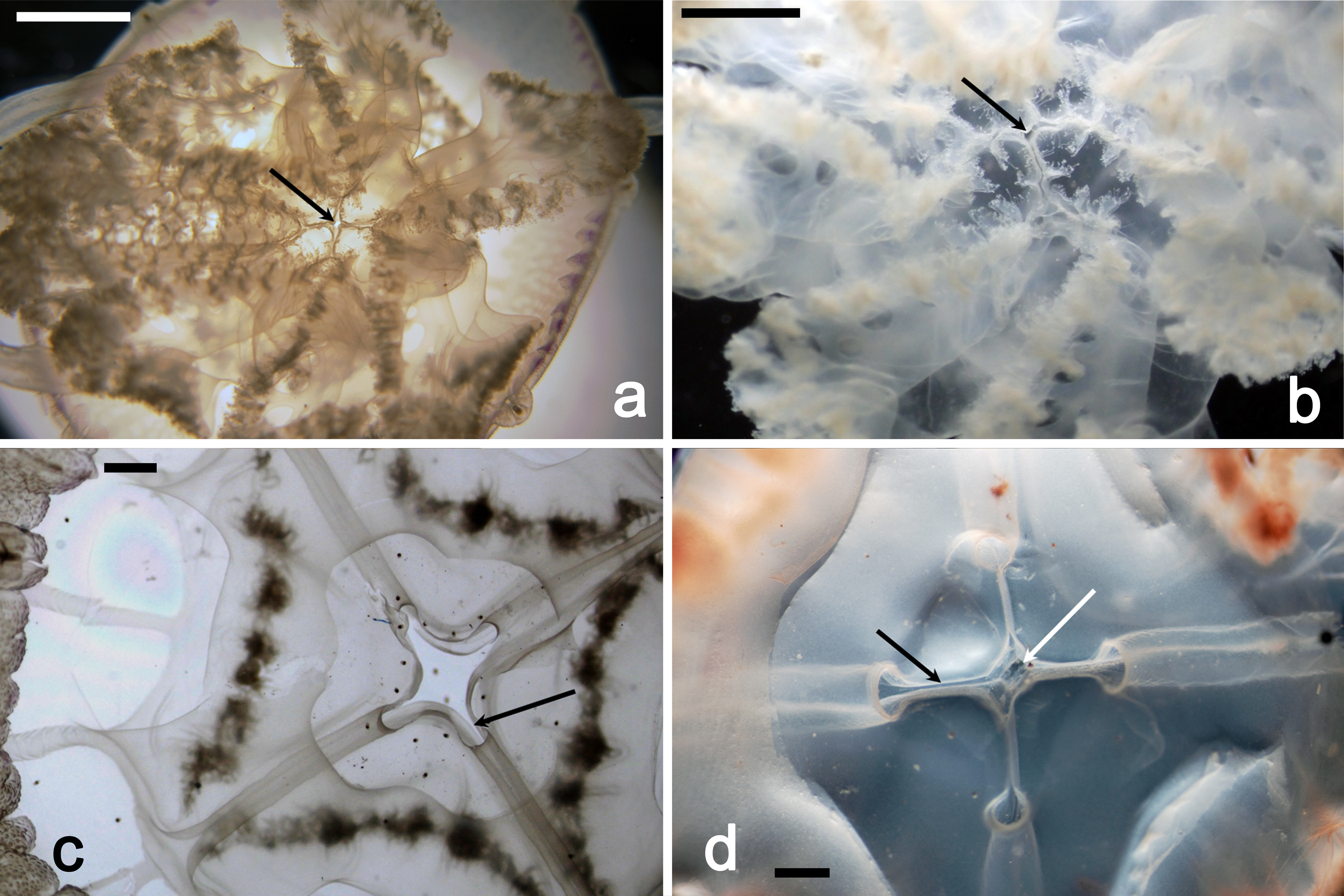

Supplement: S2 Fig — (a) Young specimen of 2.7 cm diam., subumbrellar view. Arrow indicates the residual of the central mouth opening still open (scale bar = 0.25 cm). (b) As in (a), specimen 3 cm diam. (scale bar = 0.25 cm). (c) Subumbrellar view of a 4 cm diam. specimen, manubrium excised under the genital sinuses. Visible the four perradial canals projecting into the still wide central canal (scale bar = 0.25 cm). (d) Same view in a specimen of 5 cm diam., with the same pattern as in (c), but with a noticeable size reduction of the central quadrangular canal (scale bar = 0.25 cm). Black arrows in (c) and (d)indicate the edges of the perradial canals, white arrows indicate the central quadrangular canal. (TIF) [file pone.0272023.s002.tif]

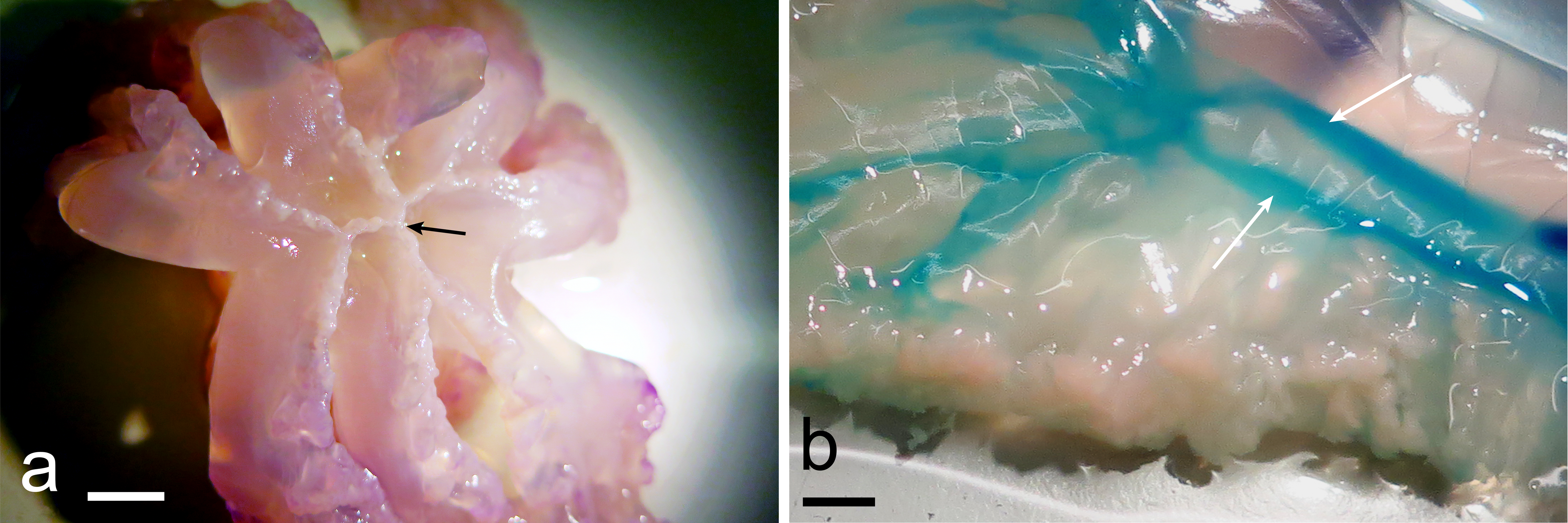

Supplement: S3 Fig — (a) Specimen of 12.4 cm diam. with the juvenile central mouth closed (arrow) (scale bar = 1 cm). (b) oral arm of a specimen of 23.5 cm diam., showing the stained hemi-canal system on the right (arrows) and on the left the anastomosis that gives rise to the canals that reach both the inner wing (bottom left) and the outer ones (top). (TIF) [file pone.0272023.s003.tif]
