# Supplementary material for: A novel endocast technique providing a 3D quantitative analysis of the gastrovascular system in Rhizostoma pulmo: An unexpected through-gut in cnidaria
Source: PLoS One. 2022 Aug 4;17(8):e0272023. doi: 10.1371/journal.pone.0272023 (PMC9352040; doi:10.1371/journal.pone.0272023)
Supplement: Attachment — Submitted filename: Response to Reviewers.docx [file pone.0272023.s011.docx]

**Reply to the Editor**

Dear E. Steele, we intend to follow your suggestion to deposit our lab. protocols in protocols.io, obviously if our work will be accepted.

The authors received no specific funding for this work.

Regarding point 4), we changed the source of satellite image contained in Fig. 12 from Google Earth to Natural Earth (Free vector and raster map data @ naturalearthdata.com). Thus, the map picture is now ok to be published.

5) We have referred Tab 2 into the text, as you asked.

All figures were checked with PACE, now all meet PLOS requirements.

Just a question. To make the separate flow regime better understandable for reviewer # 2, apart from the explanations in this "Response to Reviewers", we have also tweaked something in our Results as well as Materials and Methods, and, in this regard, we have inserted a sentence (p 14 in Results, and p 22 in Materials and Methods) on one of the experiments carried out, which was omitted in the previous version, as it was not possible to document it with videos. It is therefore a question of personal visual observations. If you think it is best to omit this piece, we would leave the decision to you.

**Reply to Reviewer #1**

Dear Jian Han, thank you for your useful comments, which we have taken into account. Now, based on your suggestions, as well as those of reviewer #2, we've extensively reviewed the through-gut discussions, both in the Abstract, Introduction and Discussion.

**Reply to Reviewer #2**

Dear reviewer #2, thank you for your comments and, in some cases, doubts. Regarding your doubts in interpreting our statements, points 1 - 3, below you will find our clarifications, and we have also revised some points in the Results, as well as in the Materials and Methods, to make our experiments and their meaning clearer, also adding a little something that was not in the previous version of the manuscript.

**1-3)** Fig 9a shows one of our first experiments with the injection of a dye solution (neutral red) into the stomach, approximately 60 sec after the injection, in exumbrellar view. We have put this picture to show how the dye diffuses in the eight adradial canals quickly reaching the inner ring canal. Fig. 9b shows the same specimen, but in lateral view, 1 min after figure 1, to highlight how the interradial canals begin to bring the dye back towards the stomach. This photo also shows how part of the dye injected into the stomach is also spreading into the outer hemi-canals of the oral arms, reaching the upper part of the external wings. Fig 9c is comparable to 9a, in a further experiment, with a different dye solution (methylene blue, has a much more intense staining capacity, and therefore the injected solution is much more diluted, and is better diffused by ciliary currents. The solution of neutral red, or that of carmine red, were much more concentrated, "oily", and diffused more slowly and with difficulty. For this reason, apart from the first couple of experiments, we used methylene blue, even if the red ones were more contrasted). The following photos are all of the specimen in 9c, in three successive stages, as in the video. These are just some of the experiments we carried out (we did multiple stain diffusion tests in summer 2019, and a further set in 2020), and as a whole they allowed us to reconstruct the flows which were then schematized in Fig.11a and 11b . Fig. 10a is the same specimen of Fig. 9a and b, used here to indicate one of the colored mucus filaments now “pushed out” from the upper openings of the external wings.

The dissection of the specimens used in the experiments, after fixation, allowed us to understand which of the hemi-canals of the oral arms were involved in the centrifugal flow, and they were always the external ones, as indicated in the scheme of Fig. 11b. But the intensity of the residual colors after fixation (after all the dyes used are vital, and therefore labile) were too weak to obtain good photos, so we proceeded to set up another set of experiments, on living parts, but dissected, of larger specimens, observable at higher magnification under a stereomicroscope. These tests have been documented in the images of Fig. 10 b-f, and related videos. These experiments have shown that: the ciliary flows, even after dissection, are maintained for a few minutes and with the same modality and directionality observed in the specimens as a whole, and as can be seen in video 6 and 7. In the movie 7, single particles present in the water, penetrate into the hemi-canals of the oral arm, both from the cut area and also from the inner wing openings (left in the video), pass in opposite directions, and those directed towards the stomach are present in the internal hemi-canal. All these data are congruent with in-sea observations, where the jellyfish, when stressed by external pressure waves caused by the movement of someone nearby, release mucus (transparent but still visible), with unpleasantly stinging characteristics, mainly from the upper and external part of the outer wings and from the distal part of the scapulae, not from the inner wings. Further observations (not included in the initial manuscript, now added) made in the summer months, during egg release events, highlighted strips of mucus, more compact, even 20 cm long and more (which soon agglutinate the phytoplankton and suspended particles present in the seawater, taking on a sandy coloration) which remain dangling from the outer wings in a relatively tenacious way, even for a long time. Also in this case these strips are located exclusively on the outer wings.

We also set up an experiment to document the flow of food collected from the oral arms in whole specimens, using living zooplankton that was filtered through a 200 μm mesh zooplankton net, and stained with a vital seawater solution of neutral red (1%). Then, medusae were fed with the stained zooplankton for a feeding time of 30 minutes, after waiting about 15 min after transferring the jellyfish to a beaker to disperse the released mucus. Unfortunately, the low density of the colored organisms, their small size, the low contrast intensity of the dye, and an unsuspected speed of transit of the single prey in the visible portion (between the end of the scapulae and the beginning of the wings) of the oral arm canals (approximately 1/2 - 1 cm/sec) did not allow us to document in an appreciable way the transit of preys inside the canals of the oral arms, difficult to focus in such a short time in a mobile jellyfish. For these reasons, this experiment was initially not reported in the manuscript. The naked eye observations, however, coincide with the data obtained in previous experiments. Now we are inserted concisely these observations.

From all these results, we had a solid basis to describe the flow, and therefore also the related transport of the preys, in the system of double canals. In the hemi-canals, at the level of the oral arms, only the internal hemi-canal showed a centripetal flow. The distinction between "mouths" and "anuses" is based on the direction of the flow, which on the basis of our data is fixed, continuous and not reversible, not even in sectioned parts.

It’s true that digestion does not take place in the manubrium, but, as already known, inside the stomach and its adjacent umbrellar canals. However, since this species feeds from filtering seawater from its openings, as well as all rhizostomeans, it is evident that internal circulation is strictly related with food ingestion and distribution.

**4)** Regarding the observation of point four, we have followed these comments, as well as the ones made by reviewer 1, and these parts are now substantially modified into the ms.

**5)** The reviewer is right, and we have no problem sharing the data obtained, on the contrary, we would be happy. Clearly, data have to be used only for scientific, educational or divulgative reasons (no lucrative use). So, we will share them freely after a simple collaboration request, with the clause of citing the sources. Now this availability has been inserted in the ms.

**6)** We agree with the reviewer that the medusozoans are an apparently incredibly heterogeneous group, from the point of view of observable body plans. But from the phylogenetic point of view they are however considered monophyletic, meaning as Medusozoa the clade Staurozoa + Cubozoa + Scyphozoa and the Hydrozoa. The so-called Myxozoa would be part of the Endocnidozoa (Myxozoa + Polypodiozoa), which, at least at the moment, are considered a sister group of the Medusozoa.

One of the several papers on this argument is:

Kayal E., Bentlage B., Pankey M.S., Ohdera A.H., Medina M., Plachetzki D.C., Collins A.G., Ryan J.F., 2018. Phylogenomics provides a robust topology of the major cnidarian lineages and insights on the origins of key organismal traits. BMC Evolutionary Biology 18:68

With the hope of having clarified your doubts, anyway we remain at your disposal for any further request for clarification, if necessary.

on behalf of all co-authors,

yours sincerely,

Massimo Avian
